# Glances at the Hospitals

**Published:** 1903-07-18

**Authors:** 


### GLANCES AT THE HOSPITALS.

### ROYAL HOSPITAL, RICHMOND, SURREY.

THE receipts for the year amounted to £4,674, and the expenditure to £4,456, showing a balance on the right side of £218; a result which is not too common in the working of our hospitals. The average number of patients in daily residence was 44; and the number of admissions during the year was 541. The average cost of each bed occupied was £77 8s. 11d.; the average cost of each patient was £6 5s. 11d., and the average cost per diem was 4s. 2d. The medical and surgical tables show no results of treatment. There is no statement referring to the anæsthetics used. A table showing the cause of death in each of the 54 cases is inserted.

### THE DONCASTER INFIRMARY.

AT this hospital the finances are not in a very good state. The committee were able to reduce the expenses by £110, but this was more than counterbalanced by a fall of £190 in the income, and there is an adverse balance at the bank larger than that in the previous year. An infirmary must rely to a very great extent on its annual subscriptions, and here these have gone down from £716 to £610. We notice that the workpeople's own subscriptions are under £240, and we think this is one direction in which improvement is possible. The cost of each in-patient was £6 8s. 10d., and the average daily cost was 3s. 6½d. At the beginning of the year 27 patients were in residence, and 248 were admitted during it, making a total of 275. Of these 182 were discharged recovered, 10 were discharged relieved, 32 were made out-patients, 21 died, and 30 remained under treatment. The medical and surgical tables merely state numbers without results, consequently they are useless for purposes of comparison. So with the 132 operations performed. We hear nothing of the results. No anæsthetic table is given.

### HUDDERSFIELD INFIRMARY.

THE expenditure has exceeded the income, and a sum of £423 is owing to the bank. The workpeople's subscriptions have increased by £136, and now amount to £1,141, which is a gratifying fact to chronicle. The Governors point out that if the employees contributed even one-halfpenny a week it would enable them to meet the requirements of the hospital; and with the facts placed prominently before them, it would be strange if the workpeople do not still further increase their subscriptions. The hospital is indebted to Mr. John Sykes, M.P., for a Finsen lamp for the treatment of lupus. There were 70 patients in the hospital at the beginning of the year, and 1,075 were admitted since then. Of the total number of 1,145 there were discharged recovered 802, relieved 146, not relieved 41, died 50, and 106 remained under treatment. The average number resident was 81. The medical and surgical tables merely state the number of patients treated for the various diseases. We do not learn much by being told that there were 17 cases of acute pneumonia, when we hear nothing of the results of treatment. The operations are, however, properly tabulated and convey the requisite information. For instance, ovariotomy was performed seven times, all being successful. The operation for appendicitis was performed three times, with a like result, and so with 12 operations for the radical cure of hernia. There is no table showing which anæsthetics were used.

